# A prospective cohort study on ultrasound simulation training in gynecology and obstetrics using pelvic, fetometry, and breast models

**DOI:** 10.1186/s12909-026-10682-0

**Published:** 2026-10-27

**Authors:** Lea Tüscher, Simone Weyers, Max Arslan, Tanja Fehm, Felix Borgmeier, Bernadette Jäger, Martina Helbig, Maggie Banys-Paluchowski, Jan Cieslik, Josefine Wadenpohl, Melissa Neubacher, Natalia Krawczyk

**Affiliations:** 1 Department of Gynecology and Obstetrics, Heinrich-Heine-University Düsseldorf, Moorenstraße 5, Düsseldorf, 40225 Germany; 2 Institute of Medical Sociology, Centre for Health and Society, Medical Faculty and University Hospital Duesseldorf, Heinrich-Heine-University Düsseldorf, Düsseldorf, Germany; 3 Simulation Center of the Department of Anaesthesiology, Heinrich-Heine-University Düsseldorf, Düsseldorf, Germany; 4 Department of Gynecology and Obstetrics, University Hospital Schleswig-Holstein Campus Lübeck, Lübeck, Germany

**Keywords:** Ultrasound simulation, Gynecology and obstetrics, Undergraduate medical education, Simulation-based learning

## Abstract

**Background:**

Ultrasound is an essential diagnostic modality in modern medicine due to its wide availability, safety, and versatility. However, its diagnostic accuracy is highly dependent on operator proficiency. Despite growing recommendations from professional societies for early and structured ultrasound education, training often begins late and predominantly in clinical practice. Simulation-based ultrasound training offers a safe and standardized learning environment, particularly for intimate examinations in gynecology and obstetrics. This study aimed to evaluate the educational effectiveness of a mandatory ultrasound simulation training integrated into an undergraduate medical curriculum.

**Methods:**

In this prospective cohort study, *n* = 143 fifth-year medical students enrolled in a mandatory obstetrics and gynecology module were allocated via a quasi-randomized approach to an intervention group or a waitlist-control group. The intervention consisted of three 20-min ultrasound simulation sessions using high-fidelity models for transvaginal sonography, fetometry, and breast ultrasound. Knowledge acquisition and self-efficacy were assessed using study-specific, pilot-tested pre- and post-intervention questionnaires. Group-level statistical analyses were performed to evaluate learning outcomes.

**Results:**

A total of 126 students completed the post-test (waitlist-control group *n* = 55; intervention group *n* = 71). Baseline characteristics and pre-test knowledge scores did not differ significantly between groups. After the intervention, the intervention group demonstrated a significantly greater increase in ultrasound knowledge compared to the waitlist-control group (*p* = .005). Participants reported high satisfaction with the training and increased self-confidence in ultrasound application. The majority (87%) expressed a desire for extended training duration.

**Conclusions:**

Mandatory ultrasound simulation training significantly improves undergraduate medical students’ ultrasound knowledge and self-confidence, even after short training interventions. These real-life evaluation data support simulation-based ultrasound education as an effective and feasible component of competency-based medical curricula.

**Supplementary Information:**

The online version contains supplementary material available at https://doi.org/10.1186/s12909-026-10682-0.

## Background

Ultrasound imaging has become an indispensable diagnostic tool in modern medicine due to its portability, non-invasive nature, absence of ionizing radiation, and applicability across a wide range of patient populations, including pregnant women and children. Its clinical value extends across numerous medical specialties; however, the diagnostic quality of ultrasound examinations remains highly dependent on the operator’s technical skills and interpretive competence [1]. Consequently, structured and early ultrasound education has been increasingly advocated by professional societies such as the German Society for Ultrasound in Medicine (DEGUM) [2].

Despite these recommendations, ultrasound training often begins only after graduation and is frequently acquired informally alongside routine clinical duties [3, 4]. This approach conflicts with the goals of competency-based medical education and may limit the development of standardized diagnostic skills among early-career physicians. In response, simulation-based ultrasound training has emerged as a promising educational strategy that allows learners to acquire foundational skills in a controlled and reproducible environment [5, 6].

Simulation-based learning is particularly favorable in gynecology and obstetrics, where the intimate nature of examinations such as transvaginal ultrasound restricts patient availability and limits opportunities for peer-to-peer or bedside teaching [6, 7]. High-fidelity simulators enable repeated practice without compromising patient comfort and have been shown to reduce cognitive load, allowing learners to focus on technical and interpretive aspects of ultrasound examinations [8]. Randomized controlled trials have demonstrated that simulation-based ultrasound training can lead to sustained improvements in clinical performance [9].

In Germany, the introduction of the National Competency-Based Catalogue of Learning Objectives for Undergraduate Medical Education (NKLM) has further accelerated the integration of ultrasound education into medical curricula [3]. Nevertheless, simulation-based ultrasound education remains an emerging field internationally, and many studies are characterized by small sample sizes and selective recruitment of motivated learners [10–16]. A notable larger comparator is the prospective GynSim study by Weimer et al. [6]: 153 fifth-year students were randomized, and 113 completed all assessments (simulation group *n* = 59; control *n* = 54). Both groups received theoretical materials, while the simulation group additionally completed 75–90 min of hands-on training. This provides an important comparator while differing from our routine-curriculum, anonymously evaluated setting.

To strengthen the scientific foundation of ultrasound simulation in undergraduate education, this study aimed to evaluate the educational impact of a mandatory, curriculum-integrated ultrasound simulation training in gynecology and obstetrics. By assessing learning outcomes in a large and heterogeneous student cohort under real-life curricular conditions, this study seeks to contribute evidence for the effectiveness and feasibility of simulation-based ultrasound education.

## Methods

### Study design and participants

This prospective cohort study employed a single-center, controlled educational intervention design with pre- and post-test assessments. The study population consisted of fifth-year medical students enrolled in a mandatory obstetrics and gynecology module at Heinrich-Heine-University Düsseldorf between July 2024 and August 2025. Attendance in the teaching unit was compulsory within the curriculum while participation in the study evaluation was voluntary and anonymous. Written informed consent was obtained from all participants prior to inclusion in the study.

**Fig. 1:**
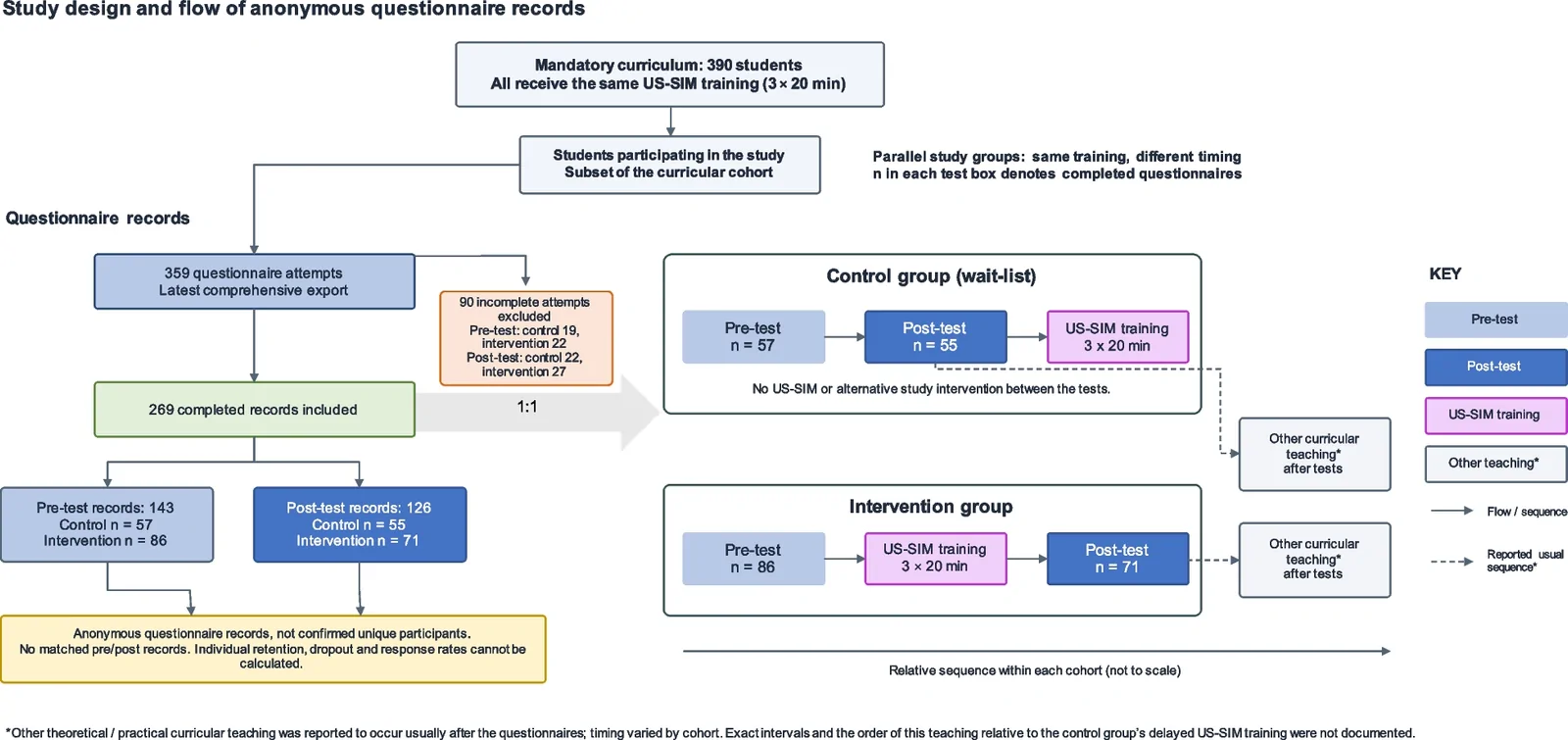
Flow of anonymous questionnaire records (left), the timeline and study design of the waitlist-control group (right)

A total of 143 students consented to participate and completed the pre-test assessment. Students were allocated via a quasi-randomized approach to either an intervention group or a control group by strictly alternating week-by-week assignment which ensured that group allocation was neutral, unbiased and independent of baseline characteristics. Furthermore, it eliminates selection bias by the study personnel and mimics the effects of true randomization (Compare Fig. 1). We employed a waitlist-control group design to ensure that all students had the opportunity to participate in the educational intervention while allowing for the comparison of learning outcomes between students who had and had not yet received the training. Both groups completed an identical pre-test before the teaching unit has begun. The intervention group completed a post-test after participating the ultrasound simulation training while the waitlist-control group passed the post-test questionnaire subsequently after the pre-test, without having undergone the training. Because the lesson is mandatory and no student group should be disadvantaged, the control group catched up the training at a later point in time and it is therefore a waitlist-control group. Ethical approval was obtained from the local ethics committee (Heinrich-Heine-University Düsseldorf ethics committee: approval number 2024–2783).

### Intervention

**Fig. 2:**
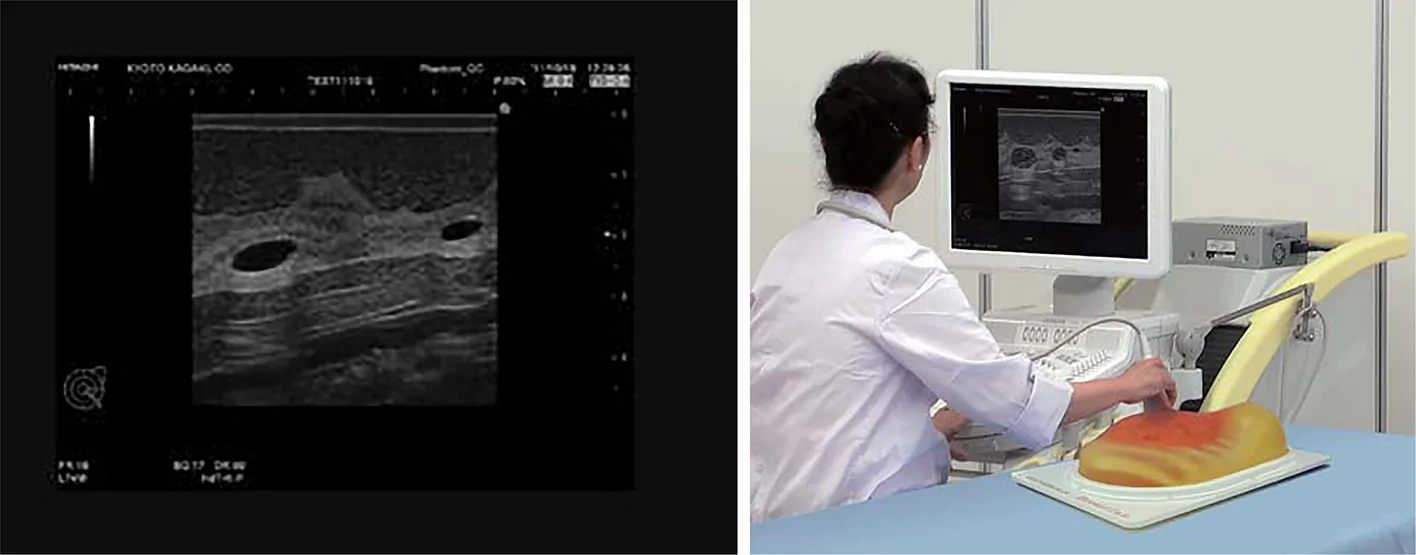
Ultrasound model of the breast (left) and ultrasound image (right) [17]. With permission of Skillsmed Germany

The intervention consisted of three ultrasound simulation sessions, each lasting 20 min, conducted under the supervision of a senior physician. Three high-fidelity simulators were used to teach transvaginal sonography (female pelvic phantom R16017, Erler Zimmer), fetal biometry (fetal model KK_US7a, Kyoto Kagaku), breast ultrasonography (Breast FAN KK_US6, Kyoto Kagaku; compare Fig. 2). All simulators were compatible with standard clinical ultrasound devices.

### Data collection

Four study-specific, pilot-tested questionnaires were administered via the SoSciSurvey platform (version 3.8.00). The pre-test forms collected demographic characteristics and ten baseline ultrasound knowledge items, while the post-tests included demographic data, a separate set of comparable knowledge score items and additional items assessing satisfaction and self-efficacy (translated questionnaire in Supplement compare Table 1). The pre- and post-test forms were not identical and contained no participant linkage code. The data collection in both questionnaires ensured the comparability of the homogenous cohorts, while the knowledge assessments served the comparison of the intervention vs. control group as well as the pre- and post-intervention skills. The knowledge assessment consisted of ten self-developed multiple-select theoretical and knowledge-application questions, including ultrasound images. To minimize guessing, an “I do not know” option was provided. Questions were scored as correct only if all response options were correctly identified. Item scores were summed to a 0–10 total. The questionnaires were pilot-tested by certified experts and board-certified physicians, achieving over 80% correctness and resulting in minor revisions.

**Table 1:** Baseline characteristics of study cohorts

**Variable**	**Control**	**Intervention**	***p*-value**^1^
	(*n* = 57 pre-test participants) (%)	(*n* = 86 pre-test participants) (%)	
**Basic characteristics (pre-test)**			
Age (years), n (%)			.242
20–25	40 (70.2%)	64 (74.4%)	
25–30	12 (21.1%)	10 (11.6%)	
> 30	5 (8.8%)	12 (14.0%)	
Gender, n (%)			.284
Female	46 (80.7%)	60 (69.8%)	
Male	11 (19.3%)	25 (29.1%)	
Diverse/no information provided	0 (0.0%)	1 (1.2%)	
Previous experienced pregnancy, n (%)			.494
Yes	1 (1.8%)	2 (2.3%)	
No	56 (98.2%)	82 (95.2%)	
No information	0 (0.0%)	2 (2.3%)	
Previous ultrasound experience, n (%)			.103
Yes (ultrasound course or internship)	51 (89.5%)	68 (79.1%)	
No	6 (10.5%)	18 (20.9%)	
Interested in the field OB/GYN, n (%)			.191
Yes	19 (33.3%)	24 (27.9%)	
No	22 (38.6%)	46 (53.5%)	
Undecided	16 (28.1%)	16 (18.6%)	

### Statistical analysis

Of the 143 students enrolled in the study, 126 completed the post-test and were included in the final analysis. Pre- and post-test data were analyzed statistically at the group level (baseline characteristics, test results and evaluation within one cohort) and were not matched individually. The primary additional comparison was the post-test total score between the intervention and control cohorts. Means, standard deviations, medians, interquartile ranges and 95% confidence intervals are reported.

Normality was assumed for the t-test comparisons reported in Table 2. Group comparisons were conducted using chi-square tests and independent t-tests, with *p* < 0.05 considered statistically significant.

**Table 2:** Test results and evaluation

**Variable**	**Control**	**Intervention**	***p*-value**^1^
**Test results (knowledge)**	**(*n* = pre-test 57/post-test 55)**	**(*n* = pre-test 86/post-test 71)**	
Pre-test score (max. 10), M (SD)	2.07 (± 1.74)	1.88 (± 1.56)	.514
Post-test score (max. 10), M (SD)	2.25 (± 1.99)	3.27 (± 1.91)	.005*
**Evaluation (Post-Test)**^**2**^	–	**(n = 71)**	
**Satisfaction and self-efficacy**			
Satisfaction with ultrasound simulation training (scale 1–5), M (SD)	–	4.42 (± 0.77)	–
Perceived benefit (scale 1–5), M (SD)	–	4.60 (± 0.65)	–
Desired training time > 1 h, %	–	87.0	–
**Self confidence in the application of**:^3^			
Transvaginal sonography, M (SD)	1.82 (± 1.07)	3.65 (± 0.81)	<.001*
Fetometry, M (SD)	1.67 (± 1.11)	3.65 (± 0.88)	<.001*
Breast sonography, M (SD)	1.71 (± 1.10)	3.58 (± 0.86)	<.001*

^*^Legend: ^1^*p*-values: Chi-Square test/t-test. *p* <.05 is considered significant ^2^ Evaluation in intervention group only (post-test). ^3^ Scale self-confidence: 1 = no, 2 = less, 3 = unchanged, 4 = some, 5 = strong self-confidence. Value > 3.0 indicates an increase in self confidence

For Table 3, post-test knowledge scores were compared between the intervention and waitlist-control groups separately within each specialty-interest category using two-sided Mann–Whitney U tests. Asymptotic p-values were calculated in Microsoft Excel with correction for tied ranks and a continuity correction of 0.5. These subgroup comparisons were exploratory; p-values were not adjusted for multiple comparisons.

**Table 3:** Test results in the category „Interested in the field “

Interested in OB/GYN (total participants)	Control (Post test)	Intervention (Post test)	*s*-value (IG vs. CG)^1^
Yes (*n* = 61)	*n* = 29 2.48 (± 2.16)	*n* = 32 3.88 (± 1.62)	.005*
Undecided (*n* = 19)	*n* = 8 2.25 (± 1.39)	*n* = 11 3.45 (± 2.25)	.235
No (*n* = 46)	*n* = 18 1.89 (± 1.97)	*n* = 28 2.50 (± 1.88)	.239

Maximum achievable score: 10. Values are presented as mean (± SD). ^1^ Two-sided Mann–Whitney U test comparing the intervention and waitlist-control groups within each interest category, using asymptotic p-values with corrections for ties and continuity. * *p* <.05; significant *p*-values are bold

Statistical analyses were performed using Microsoft Excel (version 16.103.4) and IBM SPSS Statistics (version 30.0.0.0) as well as Python 3.12 using NumPy and SciPy 1.18.1.

## Results

A total of 143 students voluntarily completed the pre-test (control group *n* = 57; intervention group *n* = 86), and 126 students completed the post-test (control group *n* = 55; intervention group *n* = 71) (Compare Fig. 1). There were no statistically significant differences between the groups respecting age, gender, prior ultrasound experience, and personal background characteristics. Most participants were aged between 20 and 25 years and female, reflecting the general demographic distribution of medical students in Germany (see Table 1).

Approximately 80% of participants reported prior ultrasound exposure through courses or internships. Roughly one third of students reported an interest in gynecology and obstetrics, with additional participants indicating uncertainty regarding future specialization.

**Fig. 3:**
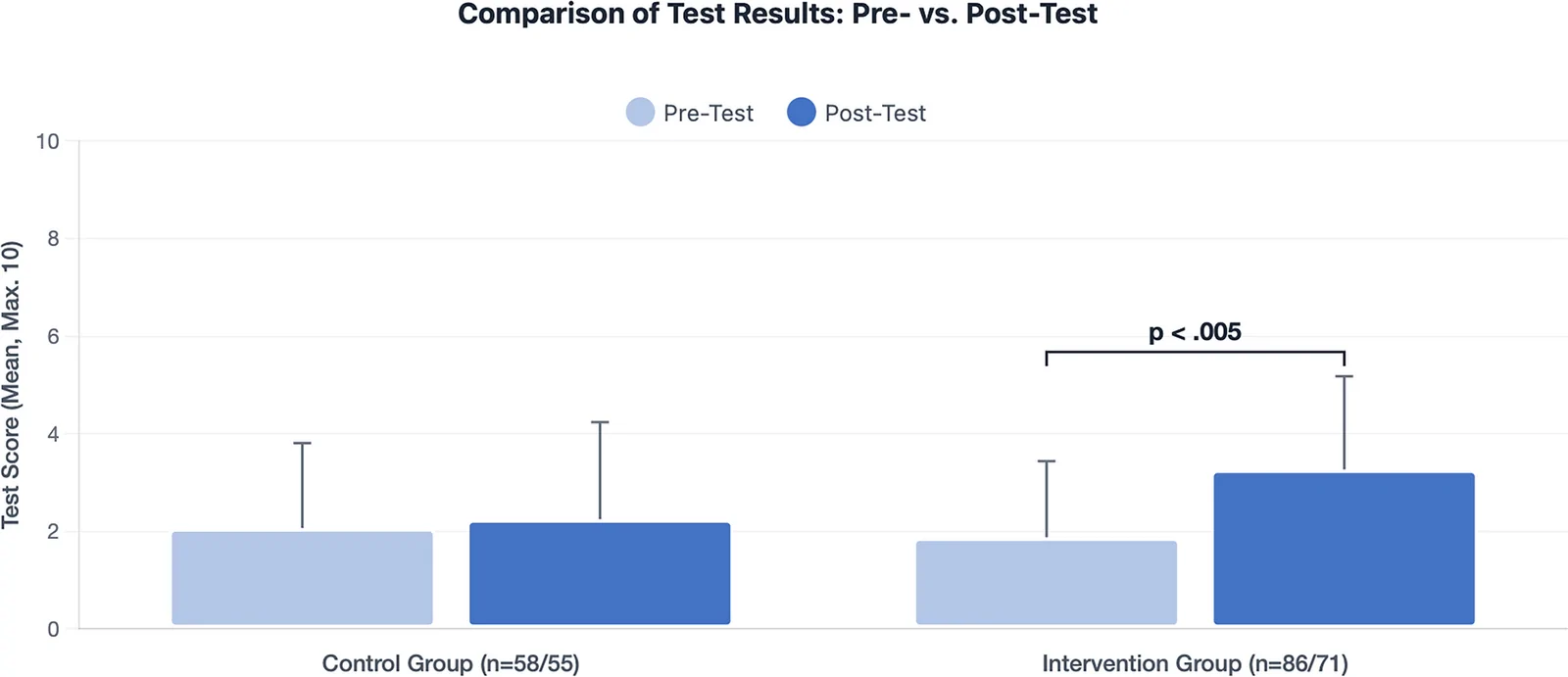
Comparison of Test Results in Control- and Intervention Group, Pre- and Posttest

Baseline knowledge scores did not differ significantly between the waitlist-control group (M = 2.07 ± 1.74) and the intervention group (M = 1.88 ± 1.56; *p* = 0.514). After the intervention, the waitlist-control group showed only a minimal, non-significant increase in knowledge (M = 2.25 ± 1.99). In contrast, the intervention group demonstrated a significant improvement, achieving a mean post-test score of 3.27 ± 1.91. The between-group difference in post-test scores was statistically significant (*p* = 0.005) and are shown in Fig. 3.

Regarding baseline characteristic, students expressing an interest in gynecology and obstetrics achieved higher mean post-test scores than undecided or uninterested peers (see Tables 2 and 3).

Post-test scores were also compared between the intervention and waitlist-control groups within each category of interest in obstetrics and gynecology (Table 3). Among students interested in the specialty, scores were higher in the intervention group (mean 3.88, SD 1.62) than in the control group (mean 2.48, SD 2.16; the Mann–Whitney U test indicated a statistically significant difference (*p* = 0.005)). The between-group differences were not statistically significant among undecided students (*p* = 0.235) or those not interested in the specialty (*p* = 0.293). These exploratory comparisons do not establish that the difference between the teaching cohorts varies by specialty interest.

Participants in the intervention group rated the ultrasound simulation training highly in terms of satisfaction (M = 4.42 ± 0.77) and perceived benefit (M = 4.60 ± 0.65). A substantial majority (87%) expressed a desire for more than one hour of training.

Self-confidence ratings indicated increased confidence in performing transvaginal sonography, fetometry, and breast ultrasound, with mean scores exceeding 3.5 on a five-point scale, reflecting a perceived improvement following the training (compare Table 2).

Participants in the intervention group rated the ultrasound simulation training highly in terms of satisfaction (M = 4.42 ± 0.77) and perceived benefit (M = 4.60 ± 0.65). A substantial majority (87%) expressed a desire for more than one hour of training.

## Discussion

This prospective cohort study demonstrates that a mandatory, simulation-based ultrasound training unit for intimate gynecological and obstetric examinations leads to measurable educational benefits in undergraduate medical education. The present data contribute to the growing body of evidence supporting ultrasound simulation as an effective and feasible teaching strategy, particularly in areas where bedside teaching is limited by ethical, logistical, or patient-related constraints.

A key finding of this study is the improvement in ultrasound knowledge observed in the intervention group after a relatively short training exposure. Three simulation units of only 20 min each resulted in statistically significant achievements of higher knowledge scores on the post test administered immediately afterward; despite comparable baseline characteristics, the waitlist-control group achieved a lower performance. It must be taken into account that this group pattern is consistent with a short-term training effect, but due to the study design, it does not allow for a definitive conclusion regarding individual or causal changes. Additionally, we did not differ between previous general ultrasound experience and special ultrasound experience in the field. Because the class is mandatory, preferences for a subject may vary. Still, participation in the study was benevolent, for that reason it is possible that the intervention group had better test results independently from personal favors for a subject or that mostly highly interested students completed the post test, because of a self-selection bias. Nevertheless, the participation rate of roughly one third of the total number of possible participants equals former rates of voluntary evaluation. The anonymous, unmatched data sets do not permit to match the results in order to make statements about individual learning outcomes. These findings may still be highly relevant in the context of increasingly condensed medical curricula and supports previous evidence that even brief, focused simulation-based interventions can yield meaningful learning effects [1, 6, 7, 11–14, 18].

While several prior studies demonstrated similar effects, many relied on longer training sessions or recruited small, highly motivated cohorts, such as residents or voluntary participants in elective courses [9, 11, 12, 14]. In contrast, the present study evaluated a short-format intervention embedded in a mandatory curriculum, thereby reflecting realistic curricular conditions. This real-life setting strengthens the external validity of the findings and suggests that ultrasound simulation does not necessarily require extensive time resources to achieve measurable improvements in knowledge. Nevertheless, the absolute post-test scores remained moderate, indicating that short interventions alone may not be sufficient to achieve advanced competence. This aligns with existing literature emphasizing the need for longitudinal curricula and repeated practice to achieve sustained and clinically transferable ultrasound skills [9, 19, 20]. The strong student demand for extended training time observed in the evaluation further supports this interpretation.

The high levels of reported satisfaction and self-confidence in this study are consistent with prior research demonstrating increased learner confidence and reception of simulation-based ultrasound education [1, 6, 7, 15, 16]. Importantly, increased self-efficacy has been associated with improved engagement and readiness for clinical learning, although it does not necessarily equate to clinical competence [9]. Therefore, simulation should be viewed as a complementary element within a broader ultrasound curriculum rather than a replacement for supervised patient-based training.

A major strength of this study lies in its evaluation of an already established, compulsory teaching format within a standard undergraduate curriculum. Unlike many educational studies conducted under optimized research conditions, this investigation reflects everyday teaching practice, including voluntary participation in evaluation and typical response rates. Approximately one third of eligible students (126 of 390 students completing the mandatory OB/GYN curriculum) consented to study participation, which is comparable to other real-life educational evaluations and similar to the participation rates reported by Weimer et al. in the GynSim study [6]. This real-life design inevitably introduces certain limitations, such as potential selection bias and non-matched pre- and post-test data. Because the curriculum was mandatory, it is possible that people interested in the field had a higher motivation to participate voluntarily in the survey, which could have caused a motivation bias. Also, we have no certainty that the identical participants have taken a pre- and a post-test because of the drop out in the post test.

The comparability is due to the cohorts with similar predictions. However, it provides valuable insight into the effectiveness of ultrasound simulation under authentic curricular conditions and illustrates how routine teaching formats can be systematically evaluated using questionnaires with different categories as baseline characteristics, knowledge items and evaluation items as part of continuous quality improvement in medical education. In this sense, the study serves as an example of education-focused quality assurance and evidence-based curriculum development. Students expressing an interest in gynecology and obstetrics achieved higher post-test scores (see Table 3), a finding consistent with previous research indicating that intrinsic motivation positively influences learning outcomes [6]. Yet, the intervention effect was observed regardless of prior ultrasound experience. The structured ultrasound-simulation teaching was new to all students, specific OB/GYN ultrasound trainings are not a fixed component of undergraduate medical curricula in Germany. This suggests that simulation-based training provides added value even for students who already had previous exposure to ultrasound during internships or courses. This supports the notion that simulator-based learning offers structured, deliberate practice that may not be consistently available in clinical settings [5, 9].

Professional societies such as DEGUM increasingly advocate for structured, competency-based ultrasound training early in medical education [2–4]. The present study supports these recommendations by demonstrating that mandatory ultrasound simulation can be implemented successfully and results in measurable learning gains, even with limited training time. Given that ultrasound skills are relevant across many medical disciplines and that physicians may encounter pregnant or gynecologic patients in various clinical contexts [18], simulation-based OB/GYN ultrasound training represents a meaningful component of a comprehensive undergraduate ultrasound curriculum.

## Conclusion

Simulation-based ultrasound training appears particularly valuable in gynecology and obstetrics, where the intimate nature of examinations such as transvaginal sonography limits patient availability and peer-to-peer teaching opportunities [6, 7]. Simulation offers a safe, standardized, and ethically sound learning environment that allows repeated practice without patient discomfort and reduces cognitive load for novice learners [8].

This study demonstrates the successful integration of a mandatory ultrasound simulation training for intimate gynecological and obstetric examinations into undergraduate medical education (see Table 2). Even short, structured simulation interventions of 20 min each led to a significant improvement in ultrasound knowledge and increased learner self-confidence. By evaluating an established teaching format under real-life curricular conditions, this study provides evidence for the educational effectiveness and feasibility of simulation-based ultrasound training and exemplifies evidence-based quality assurance in medical education. Simulation-based ultrasound training should be considered a key element in the continued development of competency-based undergraduate curricula, in line with national and professional recommendations of the DEGUM and the reforms taking place in German medical studies. As ultrasound is being extended more and more in the clinical future, a scientifical approach of the implementation in medical education can contribute to improve its application away from unstructured learning by practicing to evidence-based quality assurance.

## Supplementary Information

Supplementary Material 1.

## Data Availability

The datasets are available from the corresponding authors on a reasonable request. A part of the data generated and analyzed during this study are published in this article.

## Abbreviations

OB/GYN: Obstetrics and Gynecology
DEGUM: German Society of Ultrasound in Medicine
US-SIM: Ultrasound Simulation
NKLM: National Competency-Based Catalogue of Learning Objectives for Undergraduate Medical Education
IG: Intervention group
CG: Control group
