# Supplementary material for: A prospective cohort study on ultrasound simulation training in gynecology and obstetrics using pelvic, fetometry, and breast models
Source: BMC Med Educ. 2026 Oct 27;26:1735. doi: 10.1186/s12909-026-10682-0 (PMC13715083; doi:10.1186/s12909-026-10682-0)
Supplement: Supplementary file 1 [file 12909_2026_10682_MOESM1_ESM.docx]

**Supplement**

Table 1 Translated Questionnaire

The following table provides the complete English translation of the Pre-Test and Post-Test questionnaire used in the cohort study “Ultrasound Simulation in Gynecology and Obstetrics.” The questionnaire was specifically developed for this study to assess baseline knowledge, post-intervention learning outcomes, and participant self-assessment. Prior to implementation, the instrument underwent an internal double peer-review process within the department to ensure content validity, clinical accuracy, and clarity of wording.

Column 1 lists each item with a unique identifier and its assignment to Pretest or Posttest. Column 2 contains the translated question text. Column 3 indicates the related figure (if applicable). Column 4 lists the answer options. Correct answers would be shown in bold if provided.

| No. + Phase | Question | Related Figure | Answer Options |
| --- | --- | --- | --- |
| **Pretest** | | | |
| Pretest-01 | Do you agree to participate in the cohort study “Ultrasound Simulation in Gynecology and Obstetrics”? | - | a. Yes b. No |
| Pretest-02 | Please specify your gender. | - | a. Female b. Male c. Diverse |
| Pretest-03 | How old are you? | - | a. 20–25 b. 25–30 c. >30 |
| Pretest-04 | Do you have (estimated) more than 10 hours of prior ultrasound experience? | - | a. Yes b. No |
| Pretest-05 | If yes: Have you already completed an ultrasound course? | - | a. Yes, as part of an ultrasound course b. Yes, during a clinical traineeship (Famulatur) c. Yes, through prior professional experience |
| Pretest-06 | Have you or your partner ever been pregnant? In this context, have you had fetometric ultrasound examinations? | - | a. Yes b. No c. I prefer not to provide this information |
| Pretest-07 | Have you completed vocational training in a medical profession? (e.g., nursing/healthcare, medical assistant (MFA), physician assistant (PA), etc.) | - | a. Yes, namely: _______ b. No |
| Pretest-08 | Are you interested in gynecology as a specialty/field of further training? | - | a. Yes b. No c. Undecided |
| **Ultrasound Knowledge Pretest** | | | |
| Pretest-09 | Which probe would you use for breast sonography?  (Multiple answers possible) | - | **a. Linear probe (linear array)** b. Convex probe (curvilinear) c. Sector probe (phased array) |
| Pretest-10 | What information can you obtain from fetometry? (Multiple answers possible) | - | **a. Placental location b. Estimated fetal weight c. Amniotic fluid volume** d. Cervical length |
| Pretest-11 | What should be shown in an abdominal plane? (Multiple answers possible) | - | a. Kidney b. Heart **c. Ribs d. Liver** |
| Pretest-12 | Which plane is shown in Figure 1? (Multiple answers possible) |   Figure 1 | a. Cardiac plane b. Pelvic plane **c. Abdominal plane** d. Thoracic plane |
| Pretest-13 | Which statement about the following Figure 2 is correct? (Multiple answers possible) |   Figure 2 | a. A tumor is visible in A. b. Ectopic/displaced breast glandular tissue is visible. **c. The image shows an axilla.** d. It is recommended to biopsy finding B. |
| Pretest-14 | Which statement about Figure 3 is correct? (Multiple answers possible) |   Figure 3 | a. Visualization of the uterus could be improved with a full bladder. **b. Endometrial thickness indicates the end of the cycle.** c. Polycystic ovaries are visible. d. Multiple fibroids are visible. |
| Pretest-15 | Which structure in Figure 4 changes in early pregnancy? (Multiple answers possible) |   Figure 4 | **a. A** b. B **c. C** d. D |
| Pretest-16 | Which statement about Figure 5 is correct? (Multiple answers possible) |   Figure 5 | a. The finding in B shows spiculated extensions. **b. Area A shows breast glandular tissue.** c. Area D should not be included in the image. d. No tumors can occur in area C. |
| Pretest-17 | Which statement about Figure 6 is correct for the following image? (Multiple answers possible) |   Figure 6 | **a. This is part of a fetometric examination.** b. A transvaginal ultrasound examination was performed. **c. A posterior placenta is visible.** d. The mother has obesity. |
| Pretest-18 | Which statement about Figure 7 is correct? (Multiple answers possible) |   Figure 7 | **a. Area A shows the intraperitoneal space.** b. The image indicates a twin gestation. **c. Asymptomatic fibroids may be located in area B.** d. Area E should be measured weekly. |
| **Posttest** | | | |
| Posttest-01 | Please specify your gender. | - | a. Female b. Male c. Diverse |
| Posttest-02 | How old are you? | - | a. 20–25 b. 25–30 c. >30 |
| Posttest-03 | Do you have (estimated) more than 10 hours of prior ultrasound experience? | - | a. Yes b. No |
| Posttest-04 | If yes: Have you already completed an ultrasound course? | - | a. Yes, as part of an ultrasound course b. Yes, during a clinical traineeship  c. Yes, through prior professional experience |
| Posttest-05 | Have you or your partner ever been pregnant? In this context, have you had fetometric ultrasound examinations? | - | a. Yes b. No c. I prefer not to provide this information |
| Posttest-06 | Have you completed vocational training in a medical profession? (e.g., nursing/healthcare, medical assistant (MFA), physician assistant (PA), etc.) | - | a. Yes, namely: _______ b. No |
| Posttest-07 | Are you interested in gynecology as a specialty/field of further training? | - | a. Yes b. No c. Undecided |
| **Ultrasound Knowledge Posttest** | | | |
| Posttest-08 | Which plane shown in Figure 8 is required for fetometric measurement? (Multiple answers possible) |   Figure 8 | a. A **b. B** **c. C** **d. D** |
| Posttest-09 | What can you diagnose using transvaginal ultrasound? (Multiple answers possible) | - | **a. Ovarian tumors b. Uterine fibroids** c. Endometrial abnormalities **d. Hydrosalpinx** |
| Posttest-10 | Which description applies to the finding shown in Figure 9? (Multiple answers possible) |   Figure 9 | **a. A cyst is visible.** b. Duct ectasia is visible. **c. A well-defined mass is visible.** d. Breast glandular tissue < 25% is visible. |
| Posttest-11 | Which structure in Figure 10 allows you to determine the patient’s menstrual cycle phase? (Multiple answers possible) |   Figure 10 | **a. A** b. B **c. C** d. D |
| Posttest-12 | Which statement applies to Figure 11? (Multiple answers possible) |   Figure 11 | **a. This is a transvaginal ultrasound image.** b. An acoustic shadow is visible in the cystic structure. c. A tumor marker should be obtained for further clarification. d. The cystic structure is cycle-dependent and typically ruptures over time. |
| Posttest-13 | Which assignment is correct for Figure 12? (Multiple answers possible) |   Figure 12 | a. Area B shows a fibroid. **b. Area C shows the gestational sac. c. Area D shows the endometrium.** d. The crown–rump length can be accurately measured in area E. |
| Posttest-14 | Which statement about Figure 13 is correct? (Multiple answers possible) |   Figure 13 | **a. Polycystic structures are visible.** b. This is the right ovary. **c. The image was obtained using vaginal sonography.** d. Free fluid is visible. |
| Posttest-15 | Which statement about Figure 14 is correct? (Multiple answers possible) |   Figure 14 | **a. Area A shows skin. b. Area B shows a focal lesion.** **c. Area C could show a duct ectasia.** d. Area D shows breast glandular tissue. |
| Posttest-16 | Which statement about Figure 15 is correct? (Multiple answers possible) |   Figure 15 | a. Edema is visible in area A. **b. Dilation of a milk duct is visible in area B.** c. A focal lesion is visible in area C. **d. A lymph node is visible in area D.** |
| Posttest-17 | Which statement about the following Figure 16 is correct? (Multiple answers possible) |   Figure 16 | a. Free fluid is visible in the pouch of Douglas. **b. The urinary bladder is visualized in area A.** **c. The uterus is anteflexed.** d. There is a high suspicion of carcinoma in area D. |
| **self-evaluation section** | | | |
| Posttest-18 | Did you consider this training program relevant? | - | a. Very relevant b. Rather relevant c. Neither/nor d. Rather not relevant e. Not relevant at all |
| Posttest-19 | For you, the training for transvaginal ultrasound examinations is: | - | a. Essential b. Important c. Neutral d. Unimportant e. Irrelevant |
| Posttest-20 | For you, the training for fetometric ultrasound examinations is: | - | a. Essential b. Important c. Neutral d. Unimportant e. Irrelevant |
| Posttest-21 | For you, the training for breast ultrasound examinations is: | - | a. Essential b. Important c. Neutral d. Unimportant e. Irrelevant |
| Posttest-22 | Overall, regarding this training, you were: | - | a. Very satisfied b. Rather satisfied c. Neither/nor d. Rather dissatisfied e. Not satisfied at all |
| Posttest-23 | At the end of this training, how do you feel about performing transvaginal ultrasound examinations? | - | a. Greater confidence b. Unchanged c. Less confidence |
| Posttest-24 | At the end of this training, how do you feel about performing fetometric ultrasound examinations? | - | a. Greater confidence b. Unchanged c. Less confidence |
| Posttest-25 | At the end of this training, how do you feel about performing breast ultrasound examinations? | - | a. Greater confidence b. Unchanged c. Less confidence |
| Posttest-26 | How much time would you like for ultrasound training on the model? | - | a. 1–3 hours b. 3–8 hours c. More than 8 hours |
| Posttest-27 | How satisfied are you overall with the ultrasound session? | - | a. Very satisfied b. Rather satisfied c. Neither/nor d. Rather dissatisfied e. Not satisfied at all |
| Posttest-28 | Free text: Do you see potential for improvement in the ultrasound teaching session? | - | Open response |
